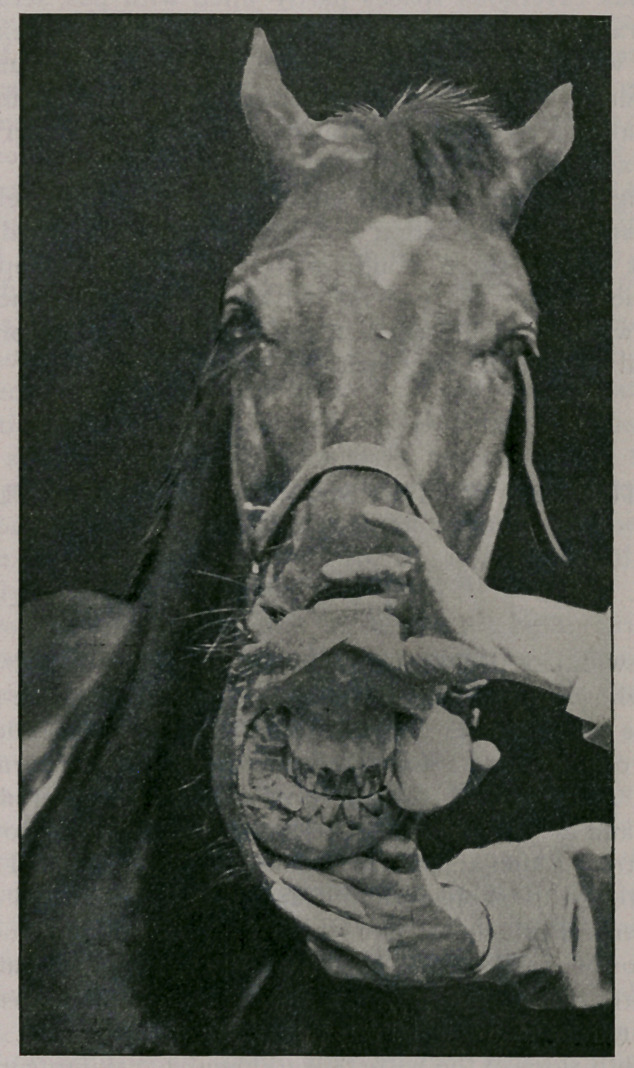# Actinomycosis of the Inferior Maxilla in a Horse

**Published:** 1896-10

**Authors:** L. E. Willyoung

**Affiliations:** Buffalo, N. Y.


### REPORTS OF CASES.

### ACTINOMYCOSIS OF THE INFERIOR MAXILLA.

By L. E. WILLYOUNG, D.V S.,
BUFFALO, N. Y.

SUBJECT: Black gelding, 15.3 hands high, about twelve years of age; general condition poor.

HISTORY: A supposed injury to the jaw one and one-half years ago. A small enlargement appeared and gradually increased in size, and had been previously diagnosed as resultant to an ulcerated tooth.

GENERAL APPEARANCE: A large spherical osseous tumor involving the interdental space and extremity of the lower maxilla, lower incisors pushed forward, all being loose, mucous membrane thickened, extending above the line of mastication, there being a furrow grooved in it produced by the lateral movements of the upper incisors while eating. Left canine tooth partially obliterated, right one entirely so. The mucous membrane presented several small ulcerated patches, some of which were continuous, with sinuses leading toward the centre, and discharging an offensive yellow, watery pus, mixed with blood and calcareous matter. Under surface of tongue ulcerated in places, and during periods of rest the tongue was usually seen hanging out of the mouth. Prehension accomplished with difficulty. Pulse and temperature, taken for five consecutive days, found to be quite normal.

Animal was destroyed June 17, 1896. The lower maxilla being removed, skin on the tumor was very thin, removed with difficulty; the same with the mucous membrane. Bone very soft in places, but still vascular. Upon longitudinal section numerous cavernous spaces partly filled with pus, serum, and degenerated bony matter, were revealed, microscopic examination of which demonstrated the presence of the ray fungus on five different slides, simply the scrapings of the ulcerated parts being taken. A careful examination of the remainder of the body was made, but no pathological lesions found.

Owing to the difficulty the animal experienced in prehension and mastication, emaciation and debility became marked during the last two or three months of its life. The accompanying illustrations show the general outline of the tumor, the upper surface with the mouth opened, and interior of the tumor, a longitudinal section showing inside of each half.

## Figures and Tables

**Figure f1:**
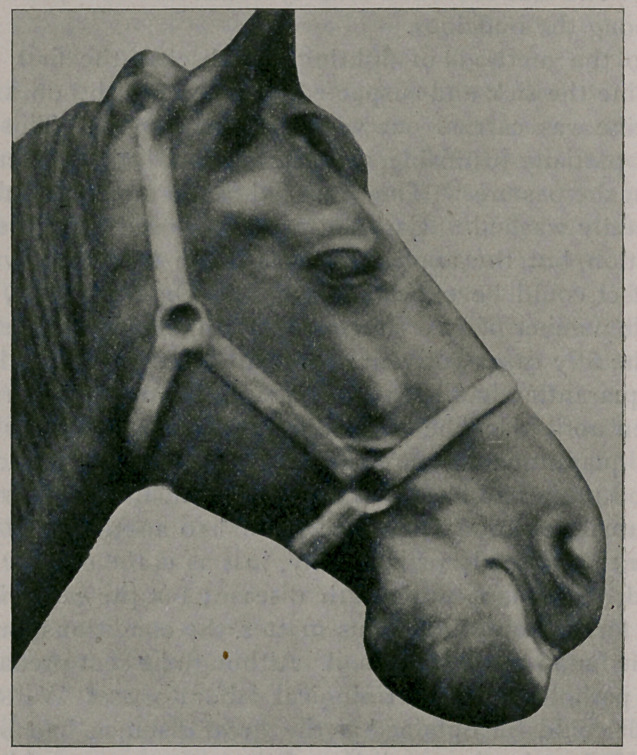


**Figure f2:**